# Recursive Paleohexaploidization Shaped the Durian Genome^1^

**DOI:** 10.1104/pp.18.00921

**Published:** 2018-11-12

**Authors:** Jinpeng Wang, Jiaqing Yuan, Jigao Yu, Fanbo Meng, Pengchuan Sun, Yuxian Li, Nanshan Yang, Zhenyi Wang, Yuxin Pan, Weina Ge, Li Wang, Jing Li, Chao Liu, Yuhao Zhao, Sainan Luo, Dongcen Ge, Xiaobo Cui, Guangdong Feng, Ziwei Wang, Lei Ji, Jun Qin, Xiuqing Li, Xiyin Wang, Zhiyan Xi

**Affiliations:** a School of Life Sciences, North China University of Science and Technology, Caofeidian District, Tangshan, Hebei, China 063210; b Center for Genomics and Computational Biology, North China University of Science and Technology, Caofeidian Dist., Tangshan, Hebei, China 063210; c Cereal and Oil Crop Institute, Hebei Academy of Agricultural and Forestry Sciences No. 162, Hengshanjie Street, Shijiazhuang, China 050035; d Fredericton Research and Development Centre, Agriculture and Agri-Food Canada, Fredericton, New Brunswick, Canada E3B 4Z7

## Abstract

The durian (*Durio zibethinus*) genome has recently become available, and analysis of this genome reveals two paleopolyploidization events previously inferred as shared with cotton (*Gossypium* spp.). Here, we reanalyzed the durian genome in comparison with other well-characterized genomes. We found that durian and cotton were actually affected by different polyploidization events: hexaploidization in durian ∼19–21 million years ago (mya) and decaploidization in cotton ∼13–14 mya. Previous interpretations of shared polyploidization events may have resulted from the elevated evolutionary rates in cotton genes due to the decaploidization and insufficient consideration of the complexity of plant genomes. The decaploidization elevated evolutionary rates of cotton genes by ∼64% compared to durian and explained a previous ∼4-fold over dating of the event. In contrast, the hexaploidization in durian did not prominently elevate gene evolutionary rates, likely due to its long generation time. Moreover, divergent evolutionary rates probably explain 98.4% of reconstructed phylogenetic trees of homologous genes being incongruent with expected topology. The findings provide further insight into the roles played by polypoidization in the evolution of genomes and genes, and they suggest revisiting existing reconstructed phylogenetic trees.

## Abstract (precis)

Durian genome reanalysis reveals a specific hexaploidization and a decaploidization in the cotton lineage that increased its evolutionary rate, explaining a previous misinterpretation.

Durian (*Durio zibethinus*), belonging to the Helicteroideae subfamily in the Malvaceae family, grows in Southeast Asia and produces fruits with a unique taste and aroma. Recently, its genome has been deciphered (Teh et al., 2017). Plant genomes often have complex genome structure, mainly due to recursive polyploidization and following genome DNA repatterning (Proost et al., 2011; Schnable et al., 2011; Jiao et al., 2012; Paterson et al., 2012). For a newly sequenced genome, comparison to its well-characterized relative plant genomes helps deconvolute its genome structure and evolutionary history (Schnable et al., 2011; Freeling et al., 2012; Murat et al., 2014; Wang et al., 2016). Before the sequencing of the durian genome, the genomes of two Malvaceae plants had been available, including cotton (*Gossypium raimondii, G. hirsutum,* and *G. raimondii*) (Li et al., 2012; Paterson et al., 2012; Wang et al., 2012) and cacao (*Theobroma cacao*) (Argout et al., 2011). Cotton was affected by recursive polyploidization events, including a core-eudicot-common hexaploidization (ECH) and a decaploidization shared by different *Gossypium* plants (Paterson et al., 2012; Wang et al., 2016). Cacao was not affected by the decaploidization, which means that a cacao (and grape, *Vitis vinifera*) genomic region would have five orthologous regions in cotton diploid (or paleodecaploid) D genome (*G. raimondii*; orthology ratio 1:5) (Wang et al., 2016).

Recently, a comparison of these three genomes showed appreciable gene synteny; and a polyploidization, previously referred to as whole-genome duplication (WGD), was proposed to have occurred during the durian evolution after the ECH. By characterizing the divergence of syntenic genes produced by the polyploidization in cotton and in durian, orthologous genes were identified between the two genomes and paralogs in each of them. A comparison of sequence divergence showed that the polyploidy-produced paralogs within cotton or durian each predated the cotton-durian orthologs. This situation was affirmed by a statistical test with posterior probability ≥ 0.9981. Therefore, the researchers proposed that durian and cotton share polyploidization events (other than ECH) that likely occurred before their divergence after ECH and places the known cotton-polyploidization also in the durian lineage. Teh et al. (2017) did not mention whether the polyploidization in cotton resulted in decaploidy and used WGD to refer to the event.

Recursive polyploidization could render unexpected complexity of plant genomes and provide enormous evolutionary forces still not fully understood (Charon et al., 2012; Kim et al., 2014; Liu et al., 2014). After each event, there could be whole-genome-level repatterning with DNA repacked into smaller numbers of chromosomes (Wang et al., 2016) and large-scale DNA losses with often only a small fraction of duplicated genes retained (Lin et al., 2014; Wang et al., 2017), possibly through a diploidization process. Fortunately, these retained hundreds of duplicates in synteny (or colinearity) often can help infer the event and let us gauge its ploidy nature, occurrence times, and dates (Bowers et al., 2003; Abrouk et al., 2010). Because of reducing selective constraint, these duplicated genes often evolve at a faster pace ( Wang et al., 2015b, 2016, 2017). Recently, a reanalysis of cucurbit genomes using a sophisticated pipeline revealed that an overlooked tetraploidization occurred ∼92–105 mya, likely having contributed to the establishment of the plant family—Cucurbitaceae—one of the largest on Earth (Wang et al., 2018). The pipeline features homologous gene dotplotting and hierarchical identification of homologous genes produced by sequential evolutionary events.

Here, we reanalyzed the durian genome using a recently proposed pipeline in comparison with other Malvaceae species (cacao and cotton [*G. raimondii*]) and the grape genome, which preserved much of the genome structure of the eudicot-common ancestor. The aim of this study was to investigate whether durian and cotton have shared or unshared polyploidization; to discover factors causing the overlook of certain paleopolyploidization; and to estimate the consequence of the overlook on polyploidization-caused changes of gene evolutionary rates on the credibility of reconstructed phylogenetic trees.

## RESULTS

### Inference of Colinear Genes

**Figure 1.**
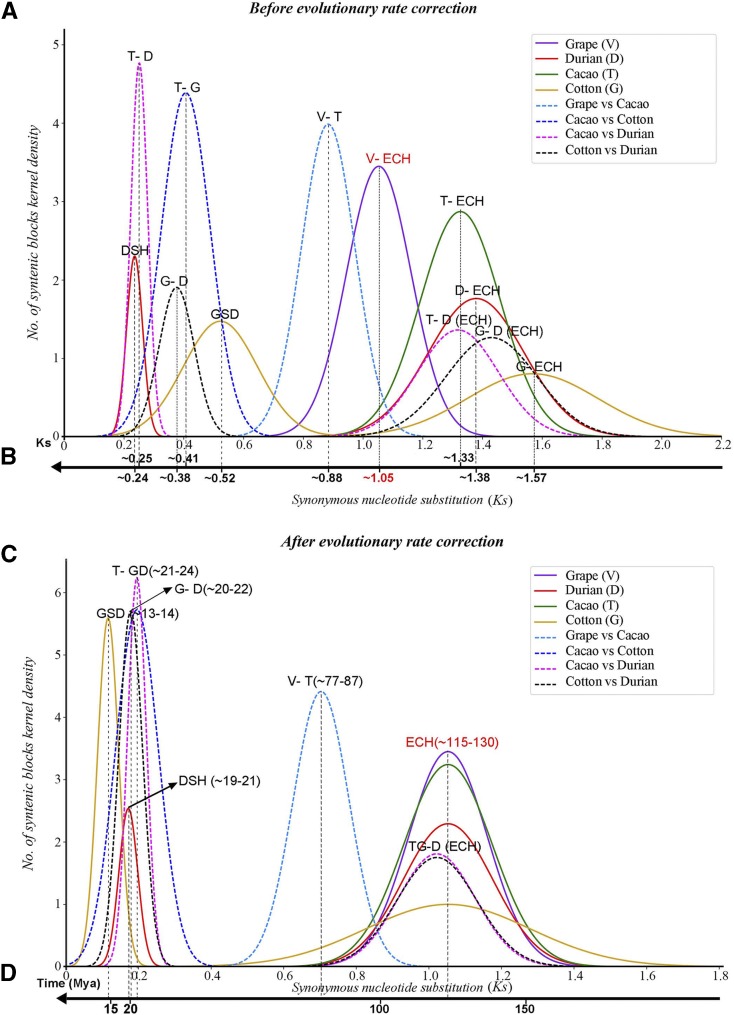
Original and corrected synonymous nucleotide substitutions (Ks) among colinear genes. Continuous lines are used to show Ks distribution in a genome, and dashed lines are among genomes. A, Distributions fitted by using original Ks values; B, Inferred means; C, Distributions fitted by using corrected Ks values; D, Inferred evolutionary dates.

By using ColinearScan, we inferred colinear genes within each genome (Supplemental Tables S1 and S2). We inferred 10,465 durian colinear gene pairs, involving 13,062 genes on 451 paralogous blocks, each having at least 4 colinear genes. We characterized the synonymous substitution divergence (Ks) between each colinear gene pair, which showed a clear bimodal structure with two distinct sets, one with Ks distribution peaking at 0.24 (±0.03) and another peaking at 1.38 (±0.16) (Fig. 1A), indicating at least two large-scale genomic duplication events. We also inferred colinear genes and characterized Ks distribution in other plant genomes (Supplemental Tables S1 and S2). The Ks distribution of cotton paralogs also showed a bimodal structure, having peaks at 0.52 (±0.12) and ∼1.57 (±0.2) (Fig. 1A; Supplemental Table S3). The peaks with larger Ks values in both durian and cotton genomes correspond to the ECH, as repeatedly reported previously (Jaillon et al., 2007; Paterson et al., 2012; Wang et al., 2016). Comparatively, the cotton event with Ks ∼0.52 was previously reported to be decaploidization (Wang et al., 2016) and seems to have occurred much earlier than the durian event with Ks ∼0.24.

We inferred colinear genes between different genomes and the corresponding Ks values (Supplemental Tables S1 and S2). For example, there were 41,958; 80,299; and 32,699 colinear genes from 2,630; 5,576; and 2,303 homologous blocks between durian and cacao, cotton, and grape, respectively. The Ks distribution between durian and each of the cacao and cotton genomes had a bimodal structure, with one corresponding to the split between two genomes and the other to the shared ECH event (Fig. 1A).

### Homologous Gene Dotplotting

**Figure 2.**
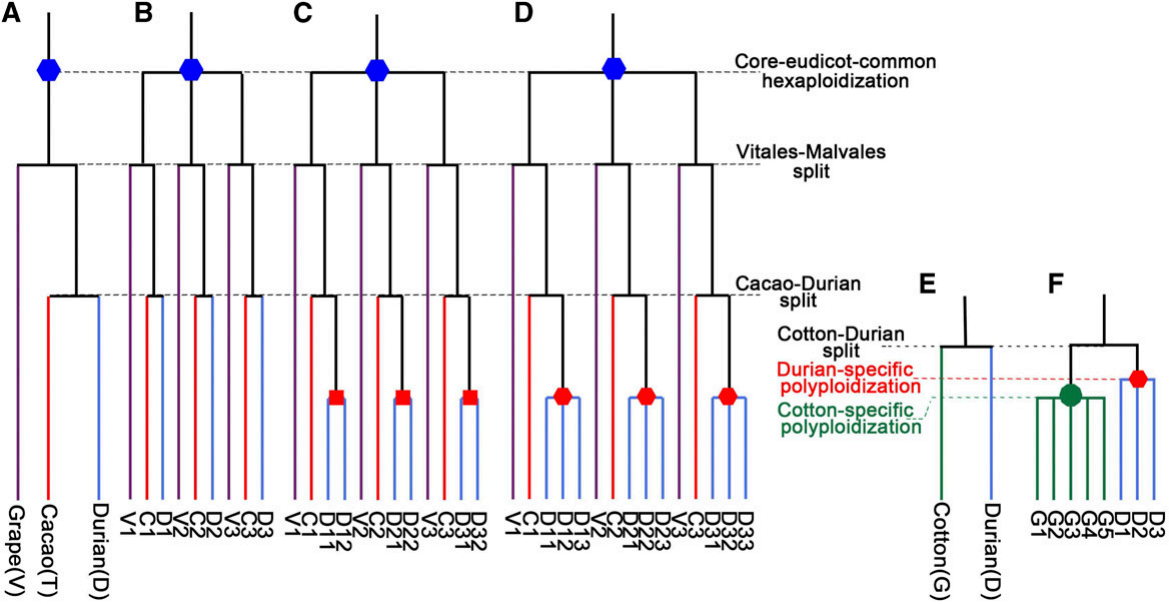
Species phylogeny and polyploidization occurrence models using haploid reference genomes. For example, in Subfig. C, the triplicated cacao paralogs, C1, C2, C3, would each have one grape ortholog and two durian orthologs. Nonorthology homologs between genomes were defined as outparalogs. Between cacao (or grape) and durian, this results in a orthology ratio 1:2 and outparalogy ratio 1:4. A, Species phylogeny; B, Assuming no durian-specific event; C, Assuming a durian-specific tetraploidization; D, Assuming a durian-specific paleohexaploidization; E, If cotton is added to the phylogeny; F, Assuming a durian-specific paleohexaploidization and a *Gossypium*-specific paleodecaploidization.

By mapping grape gene sequences onto the durian genome using a basic local alignment search tool (BLAST), we constructed the homologous dotplot by running genomic dotplotting software. Among the detected homologous genes, orthologs or paralogs were determined jointly by BLAST identity and dotplot status. The ratio of the number of probe genes to the number of targeted orthologous genes was defined as the orthology ratio. The ratio of the number of probe genes to the number of targeted outparalogous genes was defined as outparalogy ratio. The rationale of using these ratios to estimate the ploidy levels is as follows: A reference genome is a haploid genome. Therefore, a hexaploid genome has three homologous sequences in the reference genome. If there is no further polyploidization in two eudicot species after the ECH, one of the three sequences in the reference genome is the ortholog and the other two are outparalogs. Therefore, if there were no further polyploidization in durian after the shared hexaploidization (i.e. the ECH), we would expect an orthology ratio 1:1 and an outparalogy ratio 1:2 when we use a grape gene sequence to dotplot with the durian reference genome. The outparalogy was established due to the shared ECH (Fig. 2, A and B). If there were a durian-specific WGD in durian, we would expect an orthology ratio 1:2 and an outparalogy ratio 1:4 (Fig. 2C). If there were a durian-specific whole-genome triplication, we would expect an orthology ratio 1:3 and an outparalogy ratio 1:6 (Fig. 2D). If orthologous region(s) were identified, a transitive paralogy between the grape chromosomes would easily help find outparalogous regions in durian. However, owing to widespread gene losses, outparalogous blocks may be much reduced in colinear gene numbers. The other variant situations of polyploidization in durian could also be inferred in a similar manner. In the meantime, inferred colinear genes were mapped onto the homologous gene dotplots with median Ks of each colinear block displayed. This method helped distinguish orthologous blocks and outparalogous blocks between different genomes, or paralogous blocks produced by recent events from the ancient ones within each genome.

**Figure 3.**
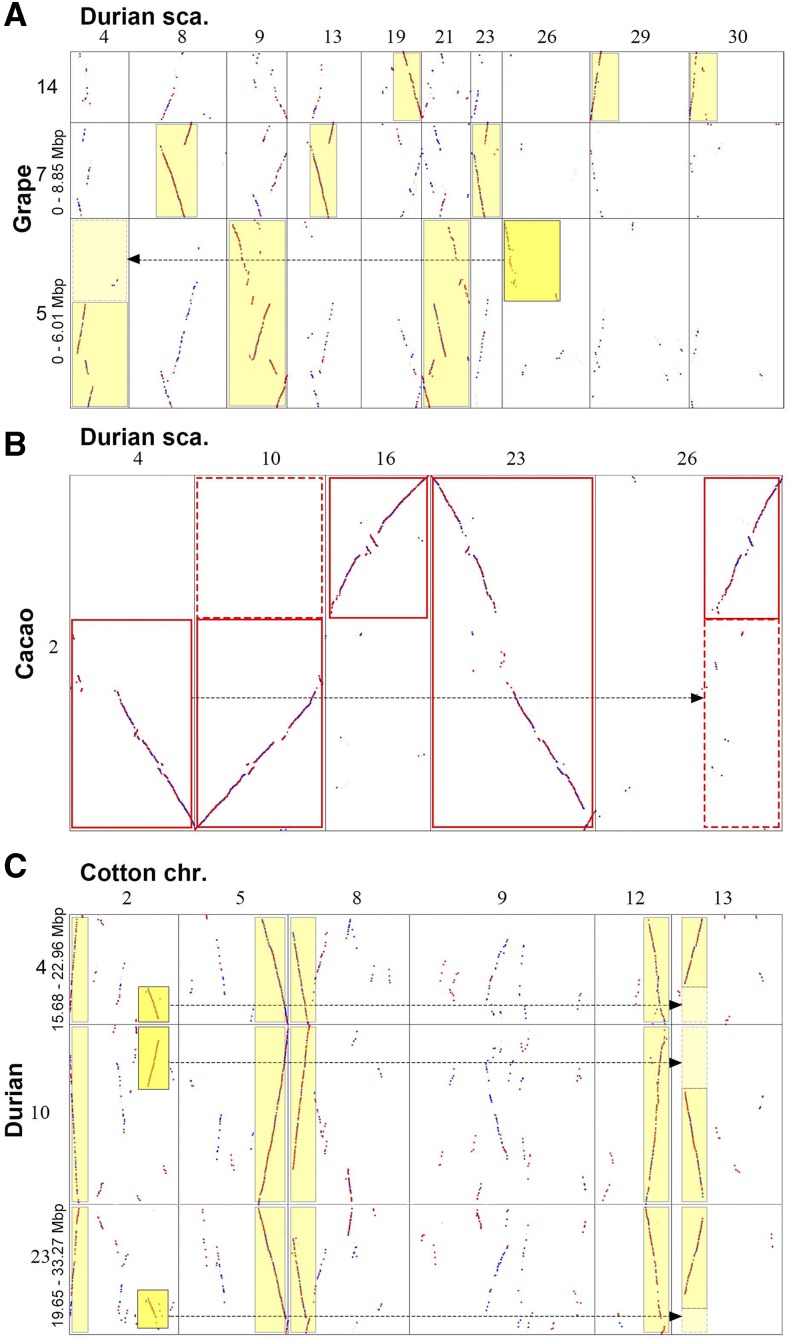
Examples of homologous gene dotplots among durian, cotton, grape, and cacao. Durian scaffold numbers and grape, cacao, and cotton chromosome numbers were shown on the tops and sides of plots, segment regions showed in megabyte (Mbp). Best-hit genes are shown in red dots, secondary hits with blue dots, and others in gray. Arrows show complement correspondence produced by chromosome breakages during evolution. A, Grape vs. durian; B, Cacao vs. durian; C, Cotton vs. durian.

The actual results that we obtained in the homologous gene dotplotting between grape and durian genomes showed an orthology ratio of 1:3 and outparalogy ratio of 1:6 (Fig. 3A; Supplemental Fig. S1). There were two distinct groups of homologous blocks, with one group with Ks values mostly <0.95, and the other group mostly >1.30. A bimodal Ks distribution shown above clearly indicated the former group was produced by orthology and the latter by the shared ECH. For the orthologous blocks, a grape chromosome region often has three independent correspondence in durian (Fig. 3A; Supplemental Fig. S1), implying an orthology ratio of 1:3. This means that a whole-genome triplication or hexaploidization occurred in the durian lineage. This durian lineage whole-genome triplication converted the then-probable “diploid” genome to a “hexaploid” genome, and this ancient after-ECH event resulted in a new hexaploidization. The peak on the Ks distribution curve for the paleodecaploidization in cotton is very sharp, suggesting that the decaploidization occurred during a relatively short period. Data in Figure 1A clearly indicate that this hexaploidization in the durian lineage is obviously not the decaploidization that occurred in the cotton lineage.

Furthermore, we drew the homologous dotplotting between durian and two Malvaceae relatives. The durian-cacao gene dotplotting showed a similar observation as in grape, with orthology ratios of 1:3 (Fig. 3B and Supplemental Fig. S2), further supporting a hexaploidization in durian. The durian-cotton (*G. raimondii*) gene dotplotting showed likely an orthology ratio of 3:5 (Fig. 2, E and F, and 3C; Supplemental Fig. S3).

In summary, the inference with three reference genomes consistently suggests a durian-specific hexaploidization (DSH), independent of a Gossypium-specific decaploidization (GSD).

### Evolutionary Rates and Dates

The GSD-produced duplicated genes had Ks values much larger than those of cotton-cacao (0.41 ± 0.08) and cotton-durian (0.38 ± 0.06) orthologs. This difference can only be explained by the possibility that cotton gene evolution became much faster after the GSD. The previous section already concluded that both GSD and DSH events occurred after the split of durian and cotton and after their common ancestor’s split from cacao. Cacao is a close outgroup of durian and cotton, and therefore we used it to evaluate the evolutionary rate difference between the two plants. The Ks distribution of cacao-durian orthologs peaked at 0.25 (±0.03), showing that after their split from cacao, cotton evolved ∼64% faster than durian.

To date the hexaploidization event in the durian lineage, we performed evolutionary rate correction to the evolutionary rates of cotton and durian duplicates (Fig. 1, C and D; Supplemental Table S4). Here, different from previous practice ( Wang et al., 2015a, 2017), we performed a two-step rate correction. In the first step, we managed to correct evolutionary rate by aligning the Ks distributions of durian, cotton, and cacao ECH duplicates to that of grape ECH duplicates, which have the smallest Ks values. However, we found the first step correction was not enough, in that the cotton rate was still elevated as compared to durian, which occurred after their split with cacao. Therefore, we performed a second step correction by aligning Ks distribution of cotton-cacao orthologs to that of durian-cacao orthologs (see Methods for details).

Eventually, we found that the DSH paralogs had a corrected Ks distribution peaking at 0.17 (±0.03). Notably, the cotton decaploidy-produced paralogs had a corrected Ks distribution peaking at 0.12 (±0.03), showing that the decaploidy was younger than the recent hexaploidization in durian. Eventually, assuming that the ECH occurred ∼115–130 mya (Vekemans et al., 2002; Jiao et al., 2012), the DSH was inferred to have occurred ∼19–21 mya, and the GSD ∼13–14 mya. In addition, the durian-cotton split was inferred to have occurred ∼20–22 mya, and they split ∼21–24 mya from cacao (Fig. 1, C and D).

### Genome Alignment and Fractionation

**Figure 4.**
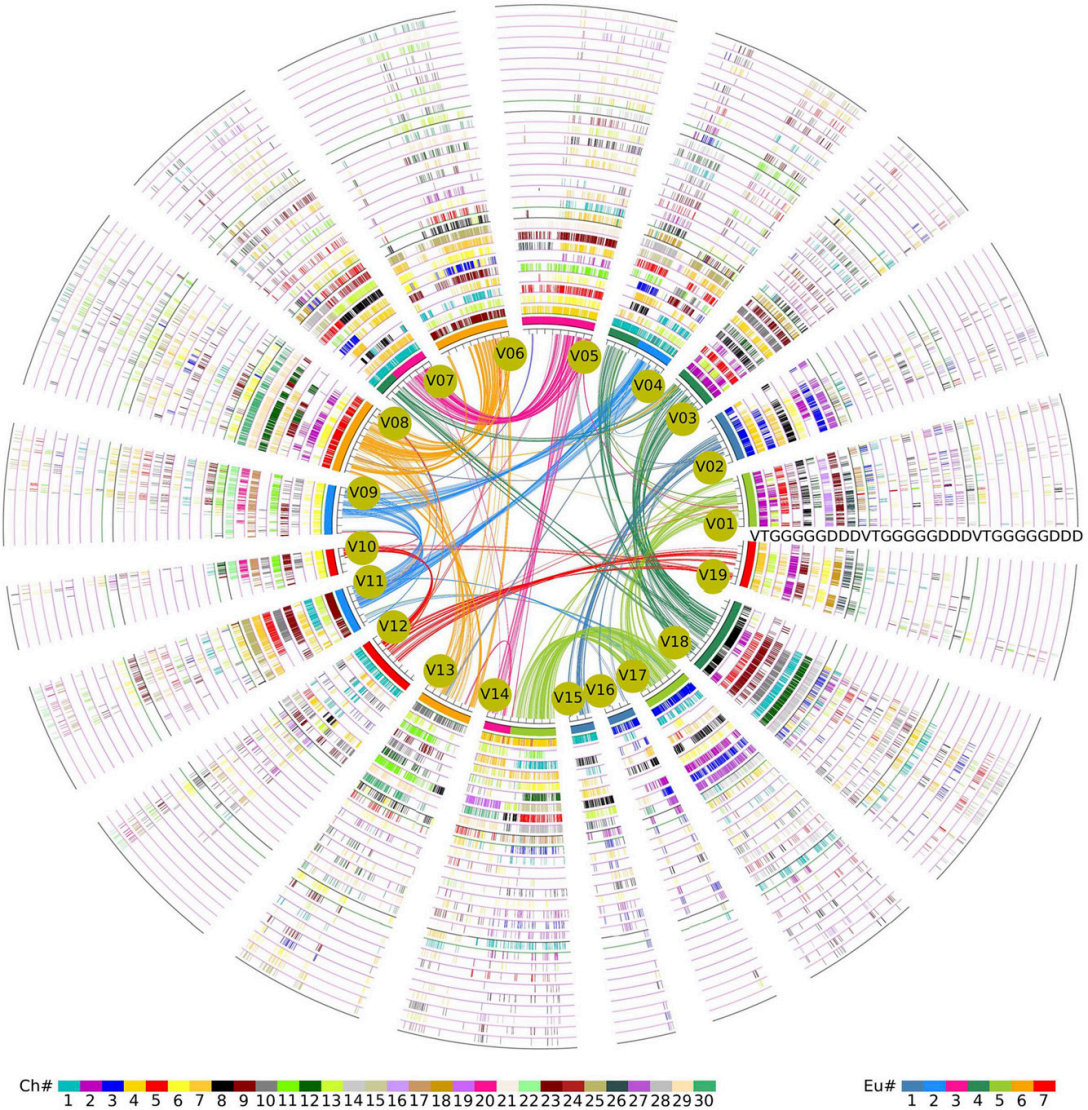
Alignment of durian, cotton, cacao, and grape genomes. The alignment was constructed by using inferred colinear genes among genomes with the grape genome as reference. The grape chromosomes form the innermost circle, and their paralogous genes in colinearity are linked curves. A grape chromosome region has 1, 5, and 3 orthologous regions in cacao, cotton, and durian genomes, respectively. The homologous regions form the other circles, displayed by short lines to show colinear genes. The short lines in grape chromosomes were colored as the 7 core-eudicot-common ancestral chromosomes inferred previously (Jaillon et al., 2007). The short lines forming chromosomes in other genomes were colored as to their respective source chromosome numbers. The color scheme is shown at the bottom. D, durian; G, cotton; V, grape .

**Figure 5.**
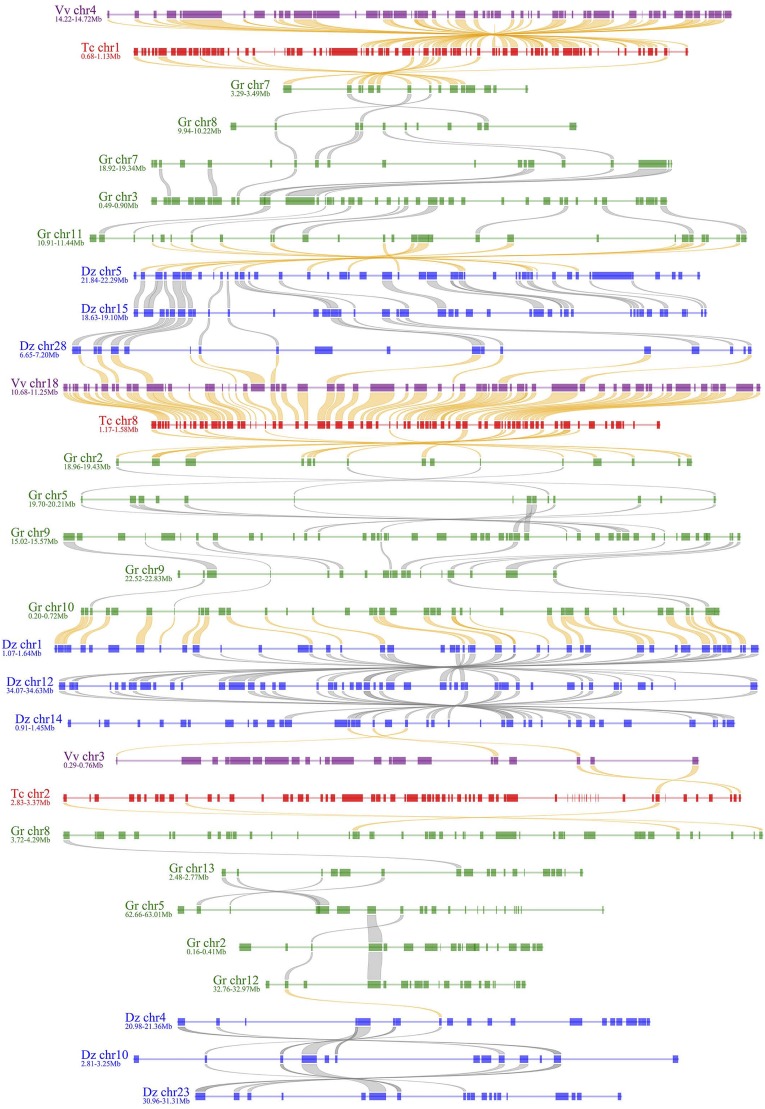
Alignment of homologous regions from durian, cotton, cacao, and grape genomes. A slice of genome-wide alignment shown in Fig. 4 is shown here in detail. This displays alignment in homologous local regions from considered genomes.

By checking homologous gene dotplot and Ks values, we separated durian paralogs produced by the ECH and the DSH. According to this analysis, the younger event created 10,367 paralogs, forming 6,181 pairs, preserved in the present genome, as compared to the ECH-produced 5,473, forming 3,299 pairs. Then, we managed to map the durian colinear genes in homologous blocks on to cacao and grape genomes. This method produced whole-genome-level alignment (Fig. 4; Supplemental Figs. S4 and S5), which showed wide-spread genomic fractionation in different genomic regions and chromosome rearrangement in each genome. A slice of the genome-level alignment can be closed displayed to highly fractionated homologous regions between genomes and in each genome (Fig. 5). As for the 32,862 cacao genes, 61% had no corresponding colinear genes in all three paralogous durian regions. We also counted missing genes in durian as compared to the other two referenced genomes. Cotton was also involved in the mapping analysis (Fig. 4; Supplemental Figs. S4 and S5).

As to the referenced 19 grape chromosomes (or 10 cacao chromosomes), we compared the genes colinear to the referenced chromosomes in tripled durian regions (dz1, dz2, dz3) and the penta-pled cotton regions (gr1, gr2, …, gr5). We checked all 3 × 5 combinations to see whether durian-cotton pair shared significantly better similarity than others. We adopted a statistic (dz × gr)/gr to measure the similarity. The best-matched regions had only an averaged similarity of 65.6%, a mere 5.8% higher than the secondary-matched regions with grape as reference (Supplemental Table S5). Similar results were shown with cacao as the reference genome (Supplemental Table S6). Grossly, this finding shows diverged colinear gene content in any compared durian and cotton homeologous regions. Moreover, this result further supports our conclusion previously mentioned that durian and cotton were independently affected by different recent polyploidization events. If the corresponding durian-cotton regions had shared an ancestor for a period after recent polyploidy, the statistics would be quite high, even near 1, if little lineage-specific gene loss occurred after their split, and much better than other combinations of comparison.

### Distorted Gene Tree Topology Due to Elevated Evolutionary Rates

**Figure 6.**
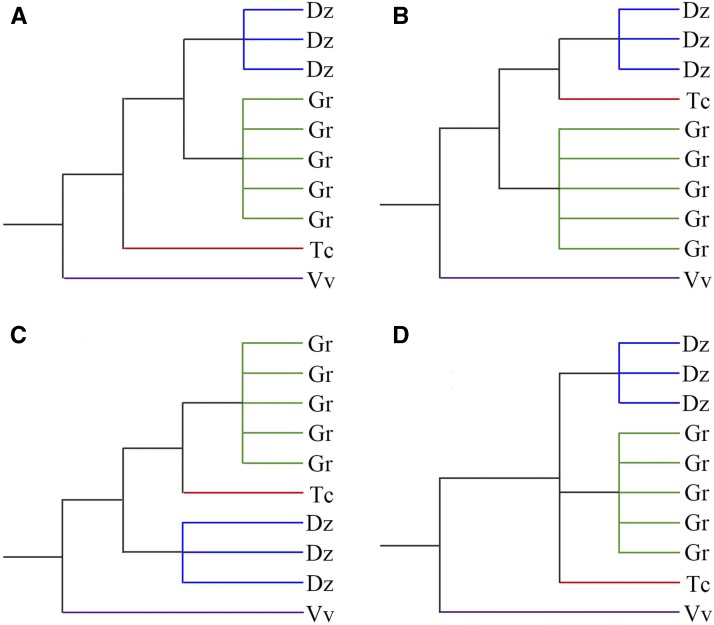
Types of reconstructed phylogeny of homologous genes. Each reconstructed tree has one grape gene as the outgroup and its cacao ortholog. The reconstructed trees are divided into four types based on the location of the cacao gene or on genes with which plant species it grouped on the phylogenetic tree. A, The tree is of expected phylogeny; B, The cacao gene is grouped with durian homologs; C, The cacao genes are grouped with cotton genes; D, The cacao gene is grouped with but not outgroup to cotton and durian genes.

We constructed evolutionary trees of homologous genes in colinear positions in the involved four genomes, and based on gene colinearity, we inferred their relationship related to speciation and polyploidization. However, we found few trees with an expected tree topology (Fig. 6). Trees were constructed for 511 groups of homologs, and each group had one grape gene as the outgroup, one cacao ortholog, at least three cotton orthologs, and at least two durian orthologs. The cacao ortholog was expected to be the outgroup of the durian and cotton orthologs. As to its actual location, the trees can be classified in to four groups (Fig. 6). Astonishingly, we found only 1.6% of constructed trees were of the expected topology, and the majority with the cacao ortholog clustered with the durian (56.9%) or the cotton orthologs (23.5%). This finding that 98.4% of reconstructed trees did not meet the evolutionary phylogeny is surely due to the elevated evolutionary rates after the polyploidization events in cotton and durian lineages. The analysis of these trees provided further evidence of separating occurrence of the DSH and GSD. Shared hexaploidization would likely result in a 1:1 correspondence between cotton and durian genes in a reconstructed phylogenetic tree of syntenic genes. In contrast, separated events, hexaploidization in durian lineage and decaploidization in cotton lineage, would likely result in cotton and durian genes forming different clusters on the tree. We checked trees constructed for cotton and durian genes with cacao and grape genes as references. Considering only branches with >70% bootstrapping percentage, we found cotton or durian genes formed clusters in 53.3% trees (270 of 507), whereas only 0.4% (2/507) had 1:1 correspondence. Besides, we used the other four approaches, including maximum likelihood, minimum evolution, UPGMA (Unweighted Pair-group Method with Arithmetic Means), and maximum parsimony, provided by using MEGA (Molecular Evolutionary Genetics Analysis), to construct 2,028 trees in total, and obtained similar results as previously with the neighbor-joining approach (separate clusters: 41.2%, 62.5%, 82.4%, and 33.3%, as compared with 0.6%, 0.8%, 1.4%, and 0.4%, respectively). Different means of tree construction resulted in consistent findings. This fact strongly supports a separating occurrence of hexaploidization and decaploidization on different lineages.

### Polyploidization Contributed to Aroma Gene Expansion

Met γ-lyase (MGL) and aminocyclopropane-1-carboxylic acid synthase (ACS) contribute to the formation of durian aroma (Teh et al., 2017). Here, we found that the two recursive hexaploidization events (both ECH and DSH) contributed to their expansion. The four MGL genes were all in colinear positions with one another. A close check of gene colinearity found that there had been one copy in the common ancestor of durian, cotton, and cacao that duplicated in the DSH to make triplicated copies in the ancestral durian genome. One of the triplicated copies produced a tandem pair. This finding means that the DSH contributed to 75% of the MGL expansion. There are 73 ACS genes in the durian genome, and 58 (70.5%) were remnants of the ECH, and 62 (84.93%) resulted from further expansion during the DSH. Comparatively, only 24 (32.9%) were ever affected by single-gene duplication after the DSH.

## DISCUSSION

Here, based on the pipeline reported previously to decipher complex genomes (Wang et al., 2018), we inferred hexaploidization in the durian lineage, independent of the decaploidization in the cotton lineage (Wang et al., 2016). The pipeline first features the production of homologous gene dotplots within a genome or between genomes. The homologous gene dotplots help distinguish orthologs from outparalogs, or separate paralogs produced by sequential polyploidization events in a genome. The homologous gene dotplotting approach was initially used to decipher recursive polyploidization in the Arabidopsis (*Arabidopsis thaliana*) genome (Bowers et al., 2003) and was adopted to understand many other complex plant genomes (Jaillon et al., 2007; Wang et al., 2011, 2016; Tomato Genome Consortium, 2012; Chalhoub et al., 2014). The pipeline also features integration of Ks between colinear genes into the homologous dotplot, providing more resolution of homologous blocks produced by different evolutionary events. A use of the pipeline revealed an overlooked tetraploidization in the common ancestor of Cucurbitaceae plants (Wang et al., 2018). The publication of the durian genome did not use homologous dotplotting to distinguish homologous regions between genomes and involving all paralogs in durian genome to estimate Ks (Teh et al., 2017). The conclusion of a shared polyploidization in durian and cotton (Teh et al., 2017) is due to overlooking the divergence in evolutionary rates, observed previously in different plants (Paterson et al., 2012; Wang et al., 2015a, 2017).

Divergent evolutionary rates of plant genes cause problems in phylogenetic and evolutionary analysis. In the grass family, it was proposed that barley (*Hordeum vulgare*), sorghum (*Sorghum bicolor*), and maize (*Zea mays*) evolved 12% to 33% faster than rice (*Oryza sativa*), which preserved the most conservative genome (Wang et al., 2015a). If assuming a common evolutionary rate of genes, this would have resulted in divergent dating of the shared tetraploidization using duplicated genes in different grasses. With grape orthologs as references, a comparison of cacao and cotton genes showed that the cotton genes evolved 19% and 15% faster than their cacao orthologs at the synonymous and nonsynonymous substitution sites, respectively (Paterson et al., 2012). The elevated evolutionary rate in cotton resulted in more divergence between cotton paralogs than their divergence from cacao orthologs. The higher evolutionary rate in cotton than in cacao (no polyploidization after split) was at least partly attributed to the occurrence of the polyploidization in cotton as elevated evolutionary rate of genes was also observed in other paleopolyploidies (Wang et al., 2011). The GSD was inferred to have occurred only ∼13–24 mya, rather than ∼59 mya as reported previously. This was likely caused by insufficient correction to Ks by referencing to the quite anciently diverged grape genes in previous analysis (Paterson et al., 2012; Wang et al., 2016). Besides, we found that, though affected by another hexaploidization after ECH, the durian evolved at similar rate or slightly faster than cacao but much slower than cotton. This finding might be because durian is a tree with a long generation time, and a long generation time may lead to reduced evolutionary rate, as observed in poplar (*Populus trichocarpa*) (Tuskan et al., 2006).

As to our further analysis, elevated evolutionary rate resulted in problematic reconstruction of phylogenetic trees. As shown previously, we inferred 98.4% of trees of durian and cotton homologs could have a topology failing to reflect their actual relationship. We tested the phylogeny reconstruction using both DNA and protein sequences, and tested different phylogenetic analysis approaches, e.g. neighbor-joining and maximal likelihood, and eventually we obtained similar error rates of tree topology (Supplemental Tables S7 and S8). This result suggests that when genes evolved at divergent rates, no matter which tree construction approach was adopted, one could not reconstruct a credible tree. Here, the inference of gene colinearity provided a precious means to infer a credible tree, showing their true relationship by relating to evolutionary events producing the homology relationship.

## MATERIALS AND METHODS

### Materials

Genome data were retrieved from public databases: durian (*Durio zibethinus*) genome (v1.0) was from GenBank (https://www.ncbi.nlm.nih.gov/genome/?term=Durian), grape (*Vitis vinifera*; v12X) and cotton D (*Gossypium raimondii*) genomes from phytozome (v2.1) (https://phytozome.jgi.doe.gov/), and cacao (*Theobroma cacao*) genome (v2) from CocogenDB (http://cacaogendb.cirad.fr/).

### Colinearity Inference

Colinear genes were inferred by using ColinearScan with maximal gap length between neighboring genes in colinearity along a chromosome region <50 genes, a setting often adopted in previous inferences (Wang et al., 2006). All the homologous blocks with ≥4 colinear gene pairs were output for further analysis (Wang et al., 2018). Putative homologous genes based on BLASTP search were used as input (E [expected] value ≤1e-5), and a relatively loose threshold here was used to help find much diverged colinear gene pairs. The significance of colinearity was tested statistically by ColinearScan.

### Homologous Gene Dotplotting

Dotplots were produced by implementing the MCSCAN (Multiple Genome Colinearity Scan) toolkit from the online database PGDD (Plant Genome Duplication Database) (http://www.plantgenome.uga.edu/pgdd/).

### Ks Calculation, Distribution Fitting, and Correction

Synonymous nucleotide substitutions on synonymous sites (Ks) were estimated by using the Nei-Gojobori approach (Nei and Gojobori, 1986) implemented using the Bioperl Statistical module.

Kernel smoothing density function **ksdensity** (width is generally set to 0.05) in MATLAB was used to estimate the probability density of each Ks list to obtain the density distribution curve. Then, Gaussian multipeak fitting of the curve was inferred by using the Gaussian approximation function **Gaussian** in the fitting toolbox **cftool**. *R^2^*, a parameter to evaluate the goodness of fit, was set to at least 95%; the smallest number of normal distributions was used to represent the complex Ks distribution; and the principle one was used to represent the corresponding evolutionary event.

To correct the evolutionary rates of ECH-produced duplicated genes, the maximum likelihood estimate *μ* from inferred Ks means of ECH-produced duplicated genes were aligned to have the same value of that of grape, which evolved the slowest. Supposing a grape duplicated gene pair to have Ks value is a random variable 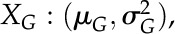 and for a duplicated gene pair in another genome the Ks to be 
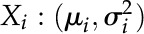
, the relative difference was



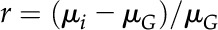



To get the corrected 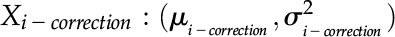, the correction coefficient was defined as



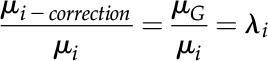



and



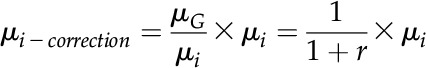



If



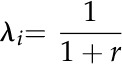



then



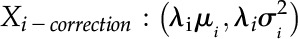



To calculate Ks of homologous gene pairs between two plants, 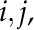 and supposing the Ks distribution was 
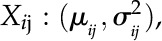
 the algebraic mean of the correction coefficients from two plants was adopted as



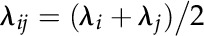



then,



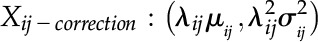



Specifically, when one the plant is grape, for the other plant, *i :*



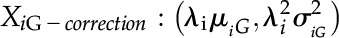



Cotton genes evolved much faster than durian genes due to paleodecaploidization. Even after a correction of Ks of ECH-produced genes, durian-cacao and cotton-cacao homologs still had very divergent distribution. Therefore, a further round of corrections was applied by aligning cotton-cacao and durian-cacao distributions to have the same distribution means. Supposing GSD duplicates had ECH-corrected Ks distribution of 
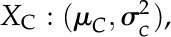
 as compared to the DSH duplicates’ Ks distribution of 
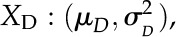
 the correction coefficient would be 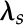. Aligning the cotton-cacao and durian-cacao ECH-corrected distributions resulted in



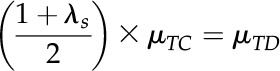





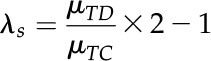



then:



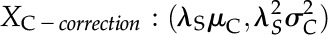



Similar to the above correction,



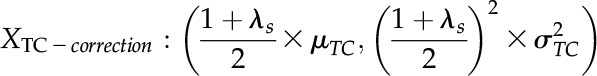



and



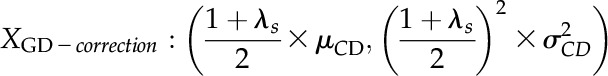





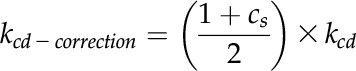



### Tree Construction

Trees of homologous genes in four genomes were constructed by implementing the maximal likelihood approach in PhyML (Guindon et al., 2005) and the neighboring-joining approach in PHYLIP using default parameter settings.

### Accession Numbers

Sequence data from this article can be found in “Materials and Methods.”

### SUPPLEMENTAL DATA

The following supplemental materials are available.

**Supplemental Figure S1.** Homologous dotplot between grape and durian genomes.
**Supplemental Figure S2.** Homologous dotplot between cacao and durian genomes.
**Supplemental Figure S3.** Homologous dotplot between cotton and durian genomes.
**Supplemental Figure S4.** Alignment of cacao and durian genomes.
**Supplemental Figure S5.** Alignment of grape and durian genomes.
**Supplemental Table S1.** Number of homologous blocks and gene pairs within a genome or between genomes.
**Supplemental Table S2.** Number of homologous genes residing in blocks within a genome or between genomes.
**Supplemental Table S3.** Kernel function analysis of Ks distribution related to duplication events within each genome and between genomes (before evolutionary rate correction).
**Supplemental Table S4.** Kernel function analysis of Ks distribution related to duplication events within each genome and between genomes (after evolutionary rate correction).
**Supplemental Table S5.** The similarity between tripled durian regions and penta-pled cotton regions with grape as reference.
**Supplemental Table S6.** The similarity between tripled durian regions and penta-pled cotton regions with cacao as reference.
**Supplemental Table S7.** The tree topology in cotton and durian genes with cacao and grape genes as references using five approaches.
**Supplemental Table S8.** The tree topology rates in cotton and durian genes with cacao and grape genes as references using five approaches.
